# Bacteriophages as an Up-and-Coming Alternative to the Use of Sulfur Dioxide in Winemaking

**DOI:** 10.3389/fmicb.2019.02931

**Published:** 2020-01-23

**Authors:** Gustavo Cordero-Bueso, Javier Moraga, María Ríos-Carrasco, Marina Ruiz-Muñoz, Jesús Manuel Cantoral

**Affiliations:** ^1^Laboratory of Microbiology, Faculty of Marine and Environmental Sciences, Department of Biomedicine, Biotechnology and Public Health, University of Cádiz, Cádiz, Spain; ^2^Laboratory of Organic Chemistry, Faculty of Sciences, Department of Organic Chemistry, University of Cádiz, Cádiz, Spain

**Keywords:** biocontrol, phages, acetic acid bacteria, lactic acid bacteria, wine safety

## Abstract

Certain acetic and lactic acid bacteria are major causes of quality defects in musts and wines, giving rise to defects such as a “vinegary,” “sharp, like nail polish-remover” taste or preventing alcoholic and/or malolactic fermentation. Sulfur dioxide is the major tool currently used in the control of these bacteria in wine. The aim of this work was to isolate bacteriophages from musts and wine of different grape varieties that were able to eliminate lactic and acetic acid bacteria spoilages at the laboratory scale. Musts obtained from grape-berries of *Vitis vinifera* cv. Chardonnay and Moscatel and a red wine made with *V. vinifera* cv. Tintilla de Rota were used to isolate bacteriophages. Bacteriophages were obtained from each of the musts and the wine and belonged to the order *Caudovirals* and the family *Tectivirals*. They were isolated by classical virology methods and identified by electron microscopy. The host bacteria used in the study were lactic acid bacteria of the species *Lactobacillus hilgardii*, *Lactobacillus plantarum*, and *Oenococcus oeni* and the acetic bacteria *Acetobacter aceti*. A comparative study was performed by adding phage titrations and SO_2_ to musts and wines, which had been previously inoculated with bacteria, to study the effectiveness of bacteriophages against bacteria. The comparative study showed that some bacteriophages were as effective as sulfur dioxide at low concentrations.

## Introduction

Bacteria are part of the natural microbiota of wine, and they play a key role in winemaking by contributing to the aroma and flavor of wine while reducing acidity. Conversely, they can cause undesirable wine spoilage problems, reducing wine quality and value (Bartowsky, 2009). Some lactic acid and acetic acid bacteria species, such as *Lactobacillus* sp., *Pediococcus* sp., and *Acetobacter* sp., are primarily responsible for the loss of quality of musts and wines, leading to defects such as a “vinegary,” “sharp, like nail polish-remover” taste or preventing alcoholic and/or malolactic fermentation (Bartowsky and Henschke, 2008; Bartowsky, 2009; Fumi et al., 2010; Sengun, 2017).

Sulfur dioxide (SO_2_) is the most commonly used antimicrobial compound in wine production due to its efficacy and low cost, being able to affect a wide variety of spoilage microorganisms. Despite being active against bacteria and some yeasts, it is innocuous to enological yeasts, since sulfur dioxide is combined with pyruvate, 2-ketoglutarate, and other compounds that are produced during alcoholic fermentation (Ough and Were, 2005). Thus, the main advantage is that *Saccharomyces cerevisiae*, the most common yeast in fermentation, is not significantly affected by sulfur dioxide at the levels used, so that it can perform the process normally (Lisanti et al., 2019). Moreover, SO_2_ protects wines against oxidation, and in adequate doses, stabilizes the color of wine and provides a complex flavor, especially in wines made with grape varieties that offer little in a sensory way (Guerrero and Cantos-Villar, 2015; Lisanti et al., 2019). The free sulfur dioxide in wine is dissociated in three chemical forms: molecular sulfite, bisulfite ion, and sulfite ion. The balance between the different chemical forms depends on the pH, the alcoholic strength, and the storage temperature of the wine. The only active form in terms of protection against contaminating microorganisms (e.g., *Brettanomyces*, *Lactobacillus*) is molecular sulfur dioxide, which is responsible for the pH value (Ough and Were, 2005). The more acidic the wine, the more active the free SO_2_ will be (Sturm et al., 2013). The initial dose depends on different factors, such as the grape maturity, the sanitary state of the grape harvest, its temperature, and, mainly, the type of wine to be obtained. Due to the great diversity of wines, there is not a pre-established dose, but winemakers adjust it according to the raw materials, technology, and products they have available.

According to Commission Regulation (EU) No. 53/2011 of January 21, 2011, amending Regulation (EC) No. 606/2009, the European Commission established an authorized total SO_2_ dose (mg/L) according to the type of wine. However, the number of people who are sensitive or allergic to this compound is increasing and, consequently, the European Commission has proposed to limit its use until its complete prohibition. This fact presents a problem for the wine industry, which may suffer major quality losses due to the uncontrolled proliferation of harmful microorganisms in wine. There has been a great deal of research on possible alternatives that can carry out the major functions of sulfur dioxide but, until now, none have been completely successful (Guerrero and Cantos-Villar, 2015). Chemical compounds such as colloidal silver complexes (Izquierdo-Cañas et al., 2012; García-Ruiz et al., 2015) and dimethyl dicarbonate (Costa et al., 2008) and even natural compounds such as lysozyme, grapevine seeds and shoots, chitosan, and bacteriocins (Lasanta et al., 2010; Garde-Cerdán et al., 2013; Liburdi et al., 2014; Dias et al., 2015; Roldán et al., 2017; Valera et al., 2017; Raposo et al., 2018; Marchante et al., 2019) are some examples. The use of polyphenolic extracts has also been investigated. Specifically, the use of enological tannins combined with lysozyme could reduce the amount of SO_2_ in wines, although the volatile composition is significantly modified (Sonni et al., 2009, 2011). In white wines, the use of almond skin and eucalyptus leaf extracts has also been proposed to reduce the SO_2_ content during barrel aging (González-Rompinelli et al., 2013).

Bacteriophages, also known as phages, are viruses that infect bacteria and promote their lysis. Recently, they have been proposed as an alternative to the use of antibiotics in animals, as biopreservers of food, and as a tool for detecting pathogenic bacteria in food processing (Mathur et al., 2003; Clark and March, 2006; Premarathne et al., 2017). The characteristics of being innocuous to human cells and specific to their host range makes bacteriophages an innovative and safe way to replace the traditional chemical compounds used in the food industry, which can generate allergies and intolerances in humans (García et al., 2008). On the other hand, the production of these chemical agents, which include pesticides and herbicides, has an environmental impact on ecosystems, besides a socioeconomic impact, due to the tendency of the population to consume more natural products with fewer allergens and additive components. There is wide recognition that bacteriophage use as an antimicrobial poses new questions regarding its eventual impact on natural environments (Meaden and Koskella, 2013). It is important to evaluate the potential risk of altering the composition of natural microbial communities through the liberation of phages from medical or agricultural applications. This disruptive effect could be harmful to ecosystems, as the phages could alter the nutrient cycle (Fernández et al., 2019). However, these changes are expected to be much less severe than with synthetic antimicrobial agents due to the high specificity of bacteriophage activity.

In this study, the aim was to search for bacteriophages that develop naturally in musts and wine. Thus, this research will allow us to identify phages able to hinder the development of the acetic and lactic acid bacteria that are responsible for quality losses in winemaking. If such viruses could be recognized as promising sulfur dioxide substitutes in combination with antioxidants, new strategies could be developed to deal with the wine spoilages caused by bacteria.

## Materials and Methods

### Strains and Culture Media

Lactic acid and acetic bacteria strains were obtained from the *Colección Española de Cultivos Tipo* (CECT). *Lactobacillus hilgardii* (CECT 4786), *Lactobacillus plantarum* (CECT 748T), and *Oenococcus oeni* (CECT 4742) were selected as lactic acid strains, while *Acetobacter aceti* (CECT 473) were chosen as the acetic bacteria.

Specific culture media were used for the growth of each strain following CECT recommendations. For lactic acid bacteria from genus *Lactobacillus*, MRS liquid/agar was used (peptone 1% w/v, meat extract 1% w/v, yeast extract 0.5 w/v, glucose 2% w/v, ammonium citrate 0.2 w/v, sodium acetate 5% w/v, K_2_HPO_4_ 2% w/v, pH 6.2–6.5). When agar was needed, 1.5% w/v (nutritive) and 0.75% w/v (layer) of agar was dissolved in 1 l of distilled water.

The *O. oeni* strain was grown in MLO medium (tryptone 1% w/v, yeast extract 0.5% w/v, glucose 1% w/v, fructose 0.5% w/v, MgSO_4_⋅7H_2_O 0, 2% w/v, MnSO_4_⋅H_2_O 0.05% w/v, diamonium citrate 0.35% w/v, L-cysteine hydrochloride 0.5% w/v, Tween 80 1 ml, pH 4.8). When agar was needed, 1.5% w/v (nutritive) and 0.75% w/v (layer) of agar was dissolved in 900 ml of distilled water.

In the case of the acetic bacteria, two culture media were used: (i) GYC medium (glucose 2% w/v, yeast extract 1% w/v, CaCO_3_ 2% w/v, pH 6.8) and (ii) Müeller-Hinton medium (meat extract 0.2% w/v, hydrolyzed casein 1.75% w/v, starch 0.15% w/v, agar 1.7% w/v, pH 7.3 ± 0.1). When agar was needed, 1.5% w/v (nutritive) and 0.75% w/v (layer) of agar was dissolved in 1 l of distilled water. The most recommended culture medium is GYC, in which the main compound is calcium carbonate, used to give opacity (Bordons and Reguant, 2013; Philippe et al., 2018). This opacity enables the bacterial growth to be monitored, as the microorganism dissolves carbonate when colonies are developed, producing holes or differently colored areas in the plate.

### Phage Plaque Assays for Titration and Isolation

#### Bacteriophage Plaque Assays

Host bacteria were grown in liquid cultures of their specific media until the exponential phase was reached. Conic tubes (Eurotubo^®^) were inoculated with 15 ml of liquid media and 1 ml of fresh bacteria culture and were incubated at 30°C for *L. hilgardii* and *L. plantarum*, 26°C for *O. oeni*, and 28°C for *A. aceti* for 24–48 h for lactic acid bacteria and from 7 to 10 days for the acetic acid baterium.

The presence of bacteriophages was determined by the double-layer agar (DLA) technique, as described by Sambrook and Russell (2001). In order to obtain the phage sample, a conic tube containing 25 ml of must or wine was centrifuged at 5000 rpm for 10 min and filtrated with a 0.2 μm filter (Millipore, MA, United States). Assays were carried out for each bacterial strain in wines and musts from three different varieties of grape: wine from *Vitis vinifera* ssp. cv. Tintilla de Rota (pH 3.53, ethanol content 14.5% v/v) and musts from *V. vinifera* ssp. cv. Moscatel (pH 4.06) and cv. Chardonnay (pH 3.88). Tenfold dilutions (from 10^–1^ to 10^–7^) of the phage samples were prepared in sterile 1× PBS solution (NaCl 138 mM, KCl 3 mM, Na_2_HPO_4_ 8.1 mM, KH_2_PO_4_ 1.5 mM). Then, 100 μl of diluted phage was added to 500 μl of the host-bacteria culture, including a control sample without phage. They were incubated for 10 min at the growth temperature of each strain. Each phage/bacteria solution was then combined with 6 ml of layer agar in a sterile 15 ml conic tube, and they were mixed briefly. Each mixture was poured onto a Petri dish containing solid nutritive agar, avoiding air bubble formation, and left to dry for 10 min in a laminar flow hood. Finally, the Petri dishes were incubated at the growth temperature of each strain for 24 h for lactic acid bacteria and from 5 to 6 days for acetic bacteria.

Plaque-Forming Units (PFU) were counted for each type of bacteria by applying the formula:

$$\frac{\text{PFU}}{\text{ml}}=\frac{{\text{n}}^{\text{o}}\,\text{plates}\,\text{×}\,\text{dilution}}{\text{volume}\,\text{of}\,\text{applied}\,\text{virus}\,\text{solution}}.$$

#### Bacteriophage Isolation

Bacteriophages were isolated from the lysis plaques formed in the Petri dishes after titration. These clear lysis zones on agar were cut with a sterile scalpel and placed in an Eppendorf microtube containing 1 ml of sterile 1× PBS solution. Three drops of chloroform were then added, and the mixture was shaken for 1 min using a vortex. It was centrifuged at 4°C at 3300 rpm and filtered with a 0.2 μm filter (Millipore, MA, United States). The process was repeated for the phage plaques obtained in each type of must and wine. The success of the isolation was checked by performing a second phage plaque assay following the same protocol as described before.

### Bacteriophage Characterization

#### Transmission Electron Microscopy (TEM)

The sample preparation consisted of negative staining. A 2% solution of fosfotungstic acid (PTA, Electron Microscopy Sciences, Hatfield, PA, United States) was made up and adjusted to pH 8.1 using normal KOH. Ten microliters of each phage suspension in PBS was added to 10 μl of PTA, and the solution was then poured onto carbon-coated 300 MESH copper grids. After 10 min, the grids were dried with filter paper and left to dry at room temperature. The preparations were examined in a TEM JEOL 2100.

#### Saturated Phenol DNA Extraction and Quantification

Phage stocks (obtained by the double-layer agar technique and recovered from the top layer) were enriched by adding 100 μl of each virus to 20 mL of each host-bacteria culture to obtain high-titer (10^10^ to 10^12^ phage/ml) phage concentrates for DNA extraction. After 16 h of incubation at the growth temperature of each host bacterium, three drops of chloroform were added, and the cultures were then centrifuged at 14000 rpm at 4°C for 30 min. The supernatant was collected and filtered with a 0.20 μm Millipore filter.

A total of 400 μl of the filtered phage suspension was added to a solution containing 20 μl of EDTA (0.5 M Ethylenediaminetetraacetic Acid, pH 8, Sigma-Aldrich), 50 μl of SDS 10% (Sodium Dodecyl Sulfate, Pronadisa, Madrid, Spain), and 5 μl of proteinase K (Panreac, Glenview, IL, United States). The sample was incubated at 37°C for 1 h. Subsequently, 400 μl of saturated phenol in 0.5 M Tris–HCl (Sigma-Aldrich) was added and the mixture was centrifuged at 13000 rpm for 4 min.

The aqueous phase was collected, and 200 μl of saturated phenol in Tris–HCl and 200 μl of phenol: chloroform:isoamyl alcohol (25:24:1) (Sigma-Aldrich) were added. They were briefly mixed and centrifuged at 13000 rpm for 4 min. The aqueous phase was transferred into a new Eppendorf microtube containing 400 μl of phenol:chloroform:isoamyl alcohol and then centrifuged at 12000 rpm for 4 min. The aqueous phase was transferred back to another 2-mL Eppendorf microtube, and 200 μl of potassium acetate (3 M, pH 7) and 600 μl of isopropanol were added. It was then centrifuged at 12000 rpm for 10 min. The supernatant was discarded, and the pellet was resuspended in 200 μl of cold ethanol 70% v/v. It was then centrifuged at 13000 rpm for 5 min, and ethanol was discarded. The pellet obtained was dehydrated at 45°C for 20 min using a Speed-Vac concentrator (Eppendorf, Germany). Finally, the pellet was resuspended in 50 μl of sterile ultrapure water. The extracted DNA concentration was measured using a Nanodrop spectrophotometer (Thermo Fisher Scientific, MA, United States) at a wavelength of 260/280.

#### Restriction Enzyme Digestion

The phage genetic material was digested with restriction enzymes in order to determine its size. The enzymes used were *Eco*RI, *Eco*RV, *Hin*fI, and *Hin*dIII, all of which were from Promega (Madison, WI, United States). Restricted DNA was electrophoresed on 0.7% (w/v) agarose gel in TAE buffer 1× (40 mM Tris-acetate, 1 mM EDTA) and visualized by UV photography after staining with ethidium bromide 5 μg/mL. The Lambda phage digested with *Hin*dIII enzyme was used as a marker.

#### Pulsed-Field Gel Electrophoresis (PFGE)

Pulsed-field gel electrophoresis consisted of taking 1 μg of non-digested viral DNA sample and heating it at 60°C for 5 min to denaturalize cohesive ends. The genetic material was then cooled in ice and quickly added into a low fusion point agarose solution (cf: 0.6%, Pronadisa). It was then loaded in an agarose gel 1% in TBE 0.5× buffer (Tris 0.89 M, EDTA 0.02 M, and boric acid 0.89 M) in order to electrophorese it.

The electrophoresis conditions were an initial pulse of 0.1 s and a final pulse of 8 s. An electric field of 0.6 V/cm was applied for 15 h at 14°C and a 120° angle. After the electrophoresis had finished, the gel was stained with ethidium bromide 5 μg/mL and visualized with an imaging system with a UV transilluminator (Biorad). The genome size was determined using the Lambda phage genome digested with the restriction enzyme *Hin*dIII as a reference.

### *In vitro* Assay of Bacteriophage Efficacy

An *in vitro* assay in a 96-well multiplate (NuncTM 96-well polystyrene conical bottom Microwell^TM^, Thermo Fisher, Denmark) was carried out in order to compare the action of the bacteriophage against host bacteria with the addition of sulfur dioxide following the procedure established by Rojo-Bezares et al. (2007) with some modifications and using the purified phages as the antimicrobial agents. Assays were done in 200 μl of the different culture media according to the bacteria used (MRS, MLO, or GYC/MH). This assay was also carried out in musts and wines at the pH values mentioned above. The initial concentration of inoculated bacteria was 10^6^ cells/ml after overnight growth in the indicated culture media. Pure bacteriophage solutions in PBS 1× were diluted from 10^–1^ to 10^–8^ UFP/mL and were co-inoculated with their host bacteria. On the other hand, sulfurous anhydrous dilutions were made from sodium metabisulfite (Sigma/Aldrich) from 100 to 450 mg/L, according to the doses recommended by Commission Regulation (EU) No. 53/2011, at pH 3.5 for *Lactobacillus* species and pH 4.5 for *O. oenis*. Blank and negative controls of infection were 200 μl of the correspondent culture media and 100 μl plus 100 μl of cells, respectively. Microplates were sealed with Breathe-Easy membranes (Sigma, Saint Louis, MI, United States) and placed into an automatic microplate reader (Multiskan go reader, Thermo Fisher Scientific, Waltham, MA, United States). The optical density of the mixture at 600 nm was read every hour (1 min of shaking just before automatic reading by the microplate reader) at the growth temperature of each host bacteria over 48–72 h. Assays were performed in triplicate, and the data obtained were processed with the software SkanIt RE for Multiskan GO 3.2. The absorbance data obtained were exported to Microsoft Excel for processing.

### Statistical Analysis

All statistical analysis was performed using SPSS software, version 25.0, and MS Excel for Windows. Data collected taken in triplicate and were analyzed using analysis of variance with Tukey’s multiple comparison test or by the non-parametric Mann–Whitney–Wilcoxon test.

## Results

### Bacteriophage Titration and Isolation

**FIGURE 1:**
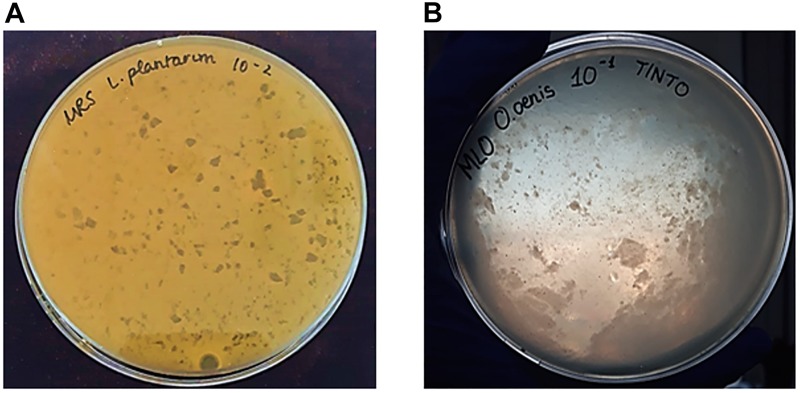
Appearance of plaques formed by bacteriophages with the double layer agar (DLA) method. **(A)** Small size and homogeneous distribution of plaques from bacteriophages with *L. plantarum* as host bacteria and isolated from Chardonnay must. **(B)** Heterogeneous plaque formed by phage infecting *O. oeni* as host bacteria, isolated from red wine (Tintilla de Rota grape variety).

Phage plaques were observed from *L. plantarum* as host bacteria from filtered samples of red wine (Tintilla de Rota) and musts (Moscatel and Chardonnay). In the case of *L. hilgardii*, bacteriophage plaques were only observed from red wine samples, while *O. oeni* showed to be infected by bacteriophages isolated from red wine and Moscatel and Chardonnay musts. Plaques from bacteriophages that infected lactic acid bacteria of genus *Lactobacillus* were small and displayed a uniform distribution (Figure 1A), while those associated with *O. oeni* showed a notable increase in the plaque size, and their distribution was very heterogeneous, occupying the entire plaque (Figure 1B).

In order to confirm the first results obtained, a second titration applying the DLA technique with purified phages isolated from the first screening was carried out. As a result, seven different bacteriophages were isolated and named according to their source, host bacteria, and plaque morphotype (Table 1). Significant differences were found between the PFU/ml in the titers made with the musts and the wine (*p* > 0.05). The number of plate-forming units (PFU) of the genus *Lactobacillus* titer was lower in red wine compared to white grape musts, which can be related to the acidity and ethanol content differences between wine and must (Table 1). As the optimum pH value for bacterial growth is near 4.5, musts formed a more suitable environment for the development of these bacterial populations and, therefore, for their predisposition to being infected by bacteriophages (Puertas, 1989; Bordons and Reguant, 2013).

**TABLE 1:** Count of plaques obtained in the second titration, applying the DLA technique.

**Host bacterium**	**Source**	**Bacteriophage**	**Lysis plaques (average ± SD)**	**PFU/mL (average ± SD)**
*L. plantarum*	Red wine	Tin1*Lp*	146 ± 5.00	2.28 ± 2.34×10^6^
	Moscatel must	Mos1Lp	190±*y*124	2.20 ± 2.10×10^11^
	Chardonnay must	Char1Lp	149 ± 90	1.10 ± 1.12×10^10^
*L. hilgardii*	Red wine	Tin1Lh	166 ± 171	1.40 ± 1.70×10^3^
*O. oeni*	Red wine	Tin1Oo	247 ± 2.00	2.00 ± 1.90×10^11^
	Moscatel must	Mos1Oo	246 ± 2.00	1.31 ± 1.34×10^14^
	Chardonnay must	Char1Oo	234 ± 4.00	1.06 ± 1.67×10^12^

*In the case of *L. hilgardii*, bacteriophage plaques were only observed from red wine samples.*

Regarding *A. aceti* titration in GYC and MH media, lysis plaques were not observed using commercial vinegar samples, red wine (Tintilla de Rota), or white musts (Moscatel and Chardonnay) as the phage isolation source.

### Characterization of the Bacteriophages

#### TEM

**FIGURE 2:**
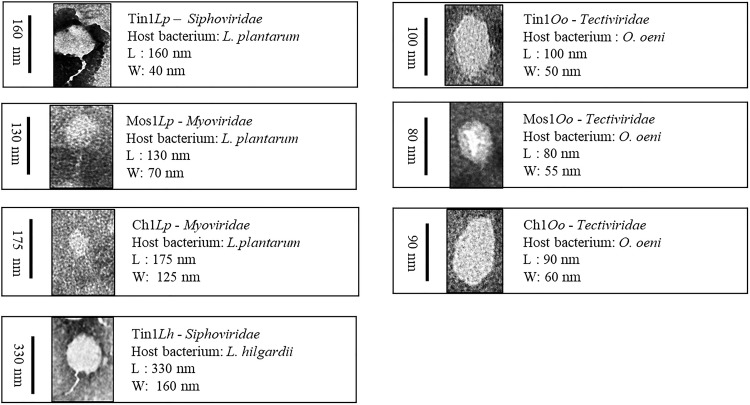
Bacteriophages were taxonomically classified according to the International Committee on Taxonomy of Viruses (ICTV). Phages that infected genus *Lactobacillus* belonged to the order *Caudovirals*, which could be classified into three families: *Myoviridae*, *Siphoviridae*, and *Podoviridae*. Those that infected *O.oeni* belonged to poliedric phages and were classified into the *Tectiviridae* family.

The bacteriophage morphology was analyzed by TEM, and they were taxonomically classified according to the International Committee on Taxonomy of Viruses (ICTV) (Ackermann, 2001). The images obtained were visually selected, and those with better resolution are featured in Figure 2.

Phages that infected genus *Lactobacillus* belonged to the order *Caudovirals*. According to Bradley’s classification (1987), three families have been described: *Myoviridae* (A), phages with a long contractile tail, *Siphoviridae* (B), phages with a long non-contractile tail, and *Podoviridae* (C), phages with a short tail (Figure 2). Each family has been divided into subgroups according to their head length: isometric (1), moderately elongated (2), and long-headed (3). Phages Tin1*Lp* and Tin1*Lh* belonged to the *Siphoviridae* (A) family, with morphotype 1 (isometric). Phages Mos1*Lp* and Char1*Lp* belonged to the *Myoviridae* (B) family, contractile tail phages, morphotype 1. On the other hand, those that infected *O.oeni* belonged to poliedric phages (D) and were classified into the *Tectiviridae* family (morphotype 4), phages with lipid vesicles and a pseudo-tail. The virus size went in decreasing order from the that which infected *L. hilgardii*, which was 330 nm in length, followed by those that infected *L. plantarum*, between 130 and 175 nm in length, and then those whose host was *O. oeni*, with a smaller size, between 80 and 100 nm length (Figure 2).

#### Genetic Material From Bacteriophages

After DNA extraction, the quantification showed high concentrations of viral genetic material (between 150 and 1000 ng/μL). The genetic material was extracted with a high purity level, as indicated by the 260/280 ratio, whose value should vary between 1.8 and 2.0. Regarding the 260/230 ratio, it is considered that DNA is pure when its value varies from 1.7 and 2, so the quality of the extracted DNA was proved.

**FIGURE 3:**
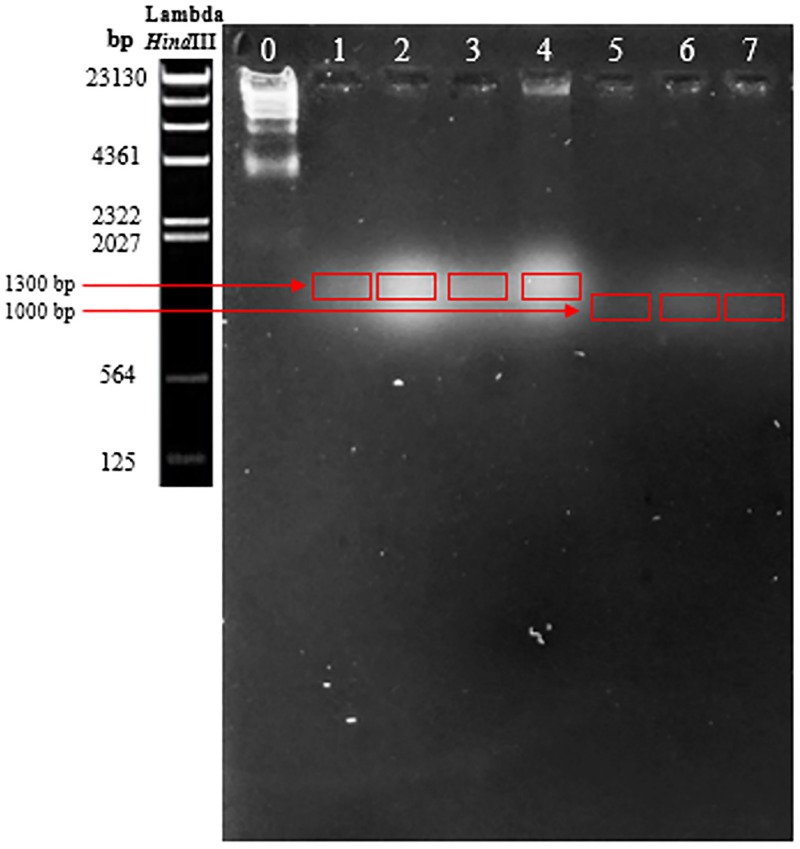
Results obtained from pulsed-field electrophoresis. Bacteriophages that infected bacteria of the genus *Lactobacillus* showed a genome approximately 1300 bp in size, while those corresponding to *O. oeni* were over 1000 bp.

The treatment with *Eco*RI, *Eco*RV, *Hin*fI, and *Hin*dIII did not digest the genomes of the phages. Several gels were made, applying different conditions, first varying the voltage intensity and then the agarose concentration used to prepare the gel. None of the tests showed a good resolution of the DNA, either the complete genome or the digested one, of any of the bacteriophage samples. However, after applying the PFGE technique, bacteriophages that infected bacteria of the genus *Lactobacillus* showed a genome of approximately 1300 bp in size, while those corresponding to *O. oeni* were over 1000 bp (Figure 3).

### *In vitro* Assay: Co-culturing Bacteriophages Against Host Bacteria Related to Different Sulfur Dioxide Doses

**FIGURE 4:**
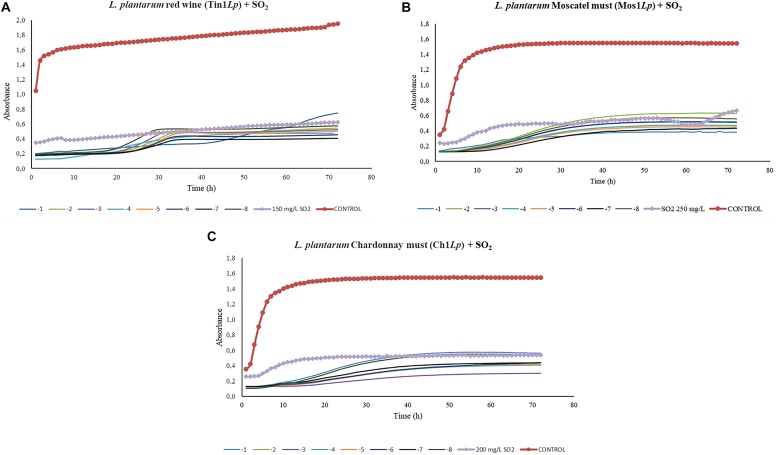
Effect of specific bacteriophages diluted from 10^– 1^ to 10^– 8^ UFP/mL on *L. plantarum* compared to sulfurous action. The viral efficacy was assessed by taking, as a reference, the host bacteria growth in red wine **(A)**, Moscatel must **(B)**, and Chardonnay must **(C)**, both in the presence of bacteriophage and of sulfur dioxide. Each point is the mean © SD of three assays.

In the growth curve corresponding to *L. plantarum*, significant differences (*p* > 0.01) were found between each dilution made from 1 × 10^–1^ to 1 × 10^–8^ and the controls in red wine and Moscatel and Chardonnay musts (Figure 4). *L. plantarum* growth values in the presence of bacteriophage were smaller than the sulfur dioxide effect when added in any concentration to the red wine (Figure 4A). This proves that the isolated bacteriophage is an effective infectious agent under the experimental conditions. Regarding Moscatel must, the growth values of *L. plantarum* with some bacteriophage dilutions were larger than or similar to the results obtained with sulfur dioxide (Figure 4B). The only favorable result obtained was 10^–1^, which was able to decrease the bacterial growth to below that with anhydride addition, which indicates that the phage Mos1*Lp* would only be efficient in controlling a certain *L. plantarum* population. Regarding Chardonnay must, a 10^–7^ concentration was enough to equate the action of sulfur dioxide, and the efficacy of the isolated bacteriophage was confirmed in all the minor dilutions tested (Figure 4C).

**FIGURE 5:**
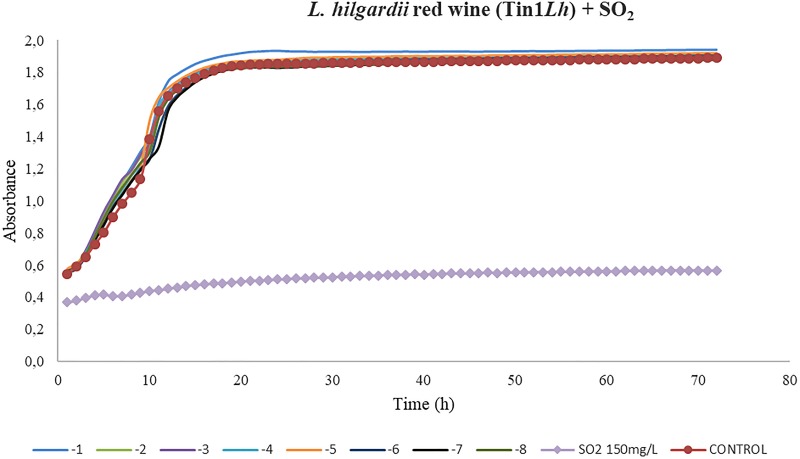
Effect of bacteriophages diluted from 10^– 1^ to 10^– 8^ UFP/mL on *L. hilgardii* compared to sulfurous action. The viral efficacy was assessed by taking, as a reference, the host bacteria growth in the presence of either bacteriophage or sulfur dioxide in red wine. Each point is the mean © SD of three assays.

In the case of the growth kinetics of *L. hilgardii*, the phage Tin1*Lh* showed an insufficient activity compared to the dose of SO_2_ for red wine (Figure 5). No significant differences between the different dilutions from 1 × 10^–1^ to 1 × 10^–8^ and the control (*p* > 0.01) were found in red wine (Figure 5). All of the bacteriophage concentrations applied (from 10^–1^ to 10^–8^ PFU/ml) followed the same trend as the control curve, so it was deduced that these concentrations lacked infective power under the experimental conditions.

**FIGURE 6:**
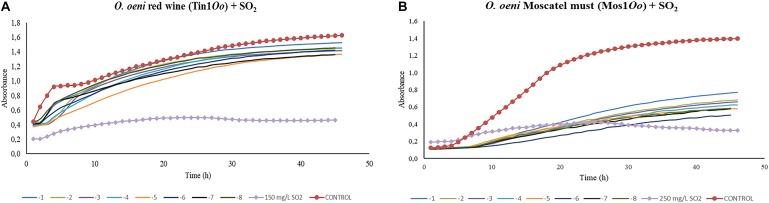
Effect of specific bacteriophages diluted from 10^-1^ to 10^– 8^ UFP/mL on *O. oeni* compared to sulfurous action. The viral efficacy was assessed by taking as a reference the host bacteria growth in red wine **(A)** and in Moscatel must **(B)**, either in presence of bacteriophage or of sulfur dioxide. Each point is the mean ± SD of three assays.

Concerning the results obtained for the kinetics of *O. oeni* in red wine, bacteriophages displayed certain inhibition activity on growth in all the dilutions applied. However, this growth decrease was not strong compared to the effect of sulfur dioxide applied in the doses used for this type of wine. Significant differences (*p* > 0.01) were found between each dilution made from 1 × 10^–1^ to 1 × 10^–8^ and the control in red wine and in Moscatel must (Figure 6). Phage samples continued to be out of range either dilution used (Figure 6A). Bacteriophages that infected *O. oeni* in Moscatel must also showed efficacy in decreasing bacterial growth (Figure 6B). Unlike red wine, there was major infective power, as the difference between the control curve and those corresponding to different dilutions was bigger. However, the kinetics graph obtained from *O. oeni* as the host bacterium in Chardonnay must did not show conclusive results data not shown. This may be due to the inability of the bacteria to grow in must, preventing the establishment of a relationship between the control growth and that associated with the phage. Consequently, it could not be compared with the sulfur dioxide effect in this grape variety must.

## Discussion

The results obtained in this work evidenced the presence of antibacterial agents in the intrinsic microbiology of the grape musts and wine used under the experimental conditions harnessed in this research. This fact was confirmed with the appearance of phage plaques in titers made for lactic acid bacteria. As stated in the results, the plaques displayed diverse morphology and distributions, which indicated the existence of different infectious agents. The number of phage plaques ranged according to the source, wine or must.

The *Lactobacillus* titer had a significantly higher PFU/ml value in white grape must than in red wine. This could be due to the ten-fold dilutions applied and to the host bacteria predisposition to grow and to be infected, as bacteriophages completely depend on their host cell, both for replicating their genome and for the production of new viral particles (Ackermann, 2003; González-García, 2014). Red wine confers a suitable environment for the growth of lactic acid bacteria because of malolactic fermentation. However, transformations produced during the alcoholic fermentation cause a pH drop and an increase in the organic acid content (Ocón, 2014). This can trigger an increase of acidity, which can lead to bacterial incompetence and, consequently, the loss of infective power of their bacteriophages. In contrast, musts have higher pH values than wine, closer to the optimum value for *Lactobacillus* to grow (pH 4.5), which could explain a greater ease of bacteriophage infection of their host. In fact, the first isolation of wine bacteriophages in 1985 by Davis et al. (1985) confirmed that pH values lower than 3.5 inhibited their growth, as verified by Henick-Kling et al. (1986a). In contrast with the results obtained in *Lactobacillus*, bacteriophages of *O. oeni* were able to infect and lyse it at red wine pH values, as stated by Henick-Kling et al. (1986b). Regarding the ethanol content in red wine used in our experiments, LAB strains (including *O. oeni*) were able to grow at 14.5% (v/v) of ethanol. Phages are usually resistant to high ethanol concentrations; Ebrecht et al. (2010) applied ethanol at different concentrations (from 10 to 100% v/v) to control the number of virulent and temperate *Lactobacillus delbrueckii* phages, and only ethanol 100% was effective against the LAB phages. In other studies, a concentration of 75% of ethanol was effective against other species of LAB phages (Binetti and Reinheimer, 2000; Suárez and Reinheimer, 2002; Quiberoni et al., 2003). Thus, the phages isolated in our study seemed to also be resistant at 14.5% of ethanol.

This study was limited, as no bacteriophages for acetic acid bacteria were isolated, though these are involved both in the production of vinegar and loss of wine quality. The acidity from the vinegar (pH < 2.8) used as a precautionary measure to avoid phages in the industrial field could be crucial to avoid the development of bacteriophages (Sellmer et al., 1992; Gullo and Giudici, 2008). Firstly, *A. aceti* showed some difficulties in growing properly. Unlike lactic acid bacteria, those from genus *Acetobacter* took more than a week to grow in the liquid medium and between 5 and 6 days in the solid one. When titrations were carried out with these cultures, changes were observed after 5 days, in contrast with the 24 h needed by bacteriophages that infected the lactic acid bacteria used in this work. Different colored areas appeared, bacteria colonies were not appreciated, that gave a first hint of the possible existence of bacteriophages. However, the use of GYC medium with calcium carbonate posed a problem for elucidating the main cause of the PFU formation, as they could be inhibition halos produced by phages or holes caused by carbonate dissolution by the bacterial metabolism while growing. As an alternative, Müeller–Hinton medium was used, since it can facilitate the observation of lysis plates because it is translucent; it has been used to show antimicrobial activity in previous acetic bacteria experiments (Du Toit and Lambrechts, 2002; Mateluna, 2006). Acetic bacteria cultures took 14 days to reach the exponential phase in the liquid medium and a week in the solid one, which makes the titre results slower than for lactic acid bacteria. These titrations did not show any lysis plate or inhibition haloes, which proved the inexistence of bacteriophages able to infect the selected strain of *A. aceti* in vinegar, wine, and musts, and allowed it to be substantiated that the plates in the GYC medium were due to the bacterial metabolism itself. However, expected results were not obtained in the titer for commercial vinegar, wine, and grape must samples. This could also be related to vinegar acid conditions or to the acidity generated by *A. aceti*. However, the existence of specific phages of acetic bacteria was documented in 1971, when Schocher et al. (1979) isolated the first bacteriophage that infected an *Acetobacter suboxidans* culture. Its host range was analyzed using 24 *Acetobacter* and *Gluconobacter* strains, where only four of the genus *Acetobacter* were susceptible to being infected. Taking into account that *A. suboxidans* turned into *Gluconobacter suboxidans* and due to the closeness of the two genuses, in 1981, Jucker and Ettlinger (1981) did a host range analysis of this phage and suggested that none of the *Acetobacter* strains but *Gluconobacter*, according to the new nomenclature, were susceptible. The presence of specific bacteriophages of acetic bacteria was also confirmed by a study of different alcohol vinegar factories in different places in Europe (France, Sweden, and Germany). Four bacteriophages were isolated and showed varied effects on the bacterial population, all of them related to fermentation breaks (Stamm et al., 1989).

Bacteriophage morphology was observed by electron microscopy. According to the ICTV guidelines, bacteriophages that infected genus *Lactobacillus* were classified as *Caudovirals* or tailed phages. Phages Tin1*Lp* and Tin1*Lh* belong to the *Siphoviridae* family, while Mos1*Lp* and Char1*Lp* belong to *Myoviridae*. Both families were obtained in the same proportion, which is in agreement with previous studies where the majority of families were *Siphoviridae* and *Myoviridae*, leaving *Podoviridae* as very rare (Ackermann, 1998). All of the phages isolated were icosahedral, which is the most frequent morphotype in *Caudovirals* (Ackermann, 2001). On the other hand, the phages that infected *O. oeni* belonged to the *Tectiviridae* family within the polyhedral phages. Although tectiviruses usually infect enteric species, bacilli, and species of the *Thermus* genus, previous studies have already demonstrated the existence of this family of viruses in wine, such as bacteriophage GC1, a new tectivirus that infects *Gluconobacter cerinus* in dry white wine-making (Ackermann and DuBow, 1987; Philippe et al., 2018).

Bacteriophage characterization was carried out using molecular microbiology techniques. Viral genetic material extraction achieved high concentrations, so it was the basis for deducing that the bacteriophages were constituted of DNA. Visualization by electronic microscopy with DNA extraction allowed the phages to be classified as double-stranded DNA, matching the majority of previous studies of wine or vinegar bacteriophages (Davis et al., 1985; Henick-Kling et al., 1986a, b; Hernández-Magadán, 2007; [Bibr B16][Bibr B49]). Despite *Eco*RI, *Eco*RV, *Hin*fI, and *Hin*dIII endonucleases having been used in similar studies of bacteriophages in dairy products and wine (Moineau et al., 1993; Ackermann, 1998; Spricigo et al., 2013), in our study, the RFLP analysis showed that these enzymes did not digest the genome of the phages isolated. It is well known that the restriction enzymes need very clean and concentrated genetic material; however, according to the extraction and quantification data obtained in our study, the concentration and ratios of DNA were sufficiently satisfactory. The phage resistance to endonuclease activity could be attributed to several factors. The most prevalent is the natural loss of restriction sites during evolution (Moineau et al., 1993; González et al., 2005), although the integration of unusual bases in the bacteriophage genome (Jensen et al., 1998; Shivu et al., 2007) and a loss of validity when there is some methylation in phage DNA affecting the restriction sites that endonucleases could recognize (Sails et al., 1998; Dini, 2011) have also been described. Sequencing and comparative genomic analysis of the bacteriophages could provide us with further information about their genetic identity.

Unlike bacteria, there are no gene sequences conserved in any of the viruses or phages that can serve as universal primer sites for Polymerase Chain Reaction (PCR) amplification. PCR-based analyses of viral DNA must therefore target specific subsets of the total viral assemblage (Steward, 2001). Taking into account the results obtained by the RFLP, we used the PFGE as a tool for bacteriophage DNA fingerprinting. This molecular technique provides a quick and visual record of the genome size distribution that can be used for qualitative and quantitative comparisons among banding patterns of the viral DNA samples (Steward, 2001). Periodic changes of electric field orientation and duration were applied, and bands evidenced the presence of DNA and gave a phage genome size estimation. According to the size range of the *Lambda* phage marker fragments digested with the restriction enzyme *Hin*dIII, phages of lactic bacteria are about 1300 bp in size, larger than those corresponding to *Oenococcus*, which approach 1000 bp. It was found that the genome of the seven isolates was much smaller than that of other lactic phages, which usually vary between 18 and 55 Kb (Jarvis et al., 1991; Moineau et al., 1993).

Regarding the assays done to evaluate the efficacy of the bacteriophages compared to that of different doses of sulfur dioxide, the most promising results were obtained in the three bacteriophages of *L. plantarum*. In the three grape varieties tested, a very low phage concentration (10^–7^) was able to substantially decrease the host-bacteria growth rate. Control curves followed a common structure, showing an initial exponential phase (initial culture) followed by a stationary phase, in which growth stabilized. In the case of red wine, the exponential phase was notably shorter than in Moscatel and Chardonnay musts. This could be due to the low availability of nutrients in fermented wine in contrast to the white musts, which contained sugars available to be used by host bacteria. In contrast to the results obtained for *L. plantarum*, the only isolated bacteriophage that infected *L. hilgardii*, Tin1*Lh*, did not show the same or comparable efficacy as the SO_2_ dose applied. Control wells, containing only bacteria, followed the same kinetic curve as all the bacteriophage doses, from 10^–1^ to 10^–8^, from which it was deduced these concentrations lacked infective power under the experimental conditions.

In general, the results of the phage titers may vary between trials, due to the resistance that can be generated by bacteria to bacteriophage infection (Ackermann, 2003). Although consideration of the influence of “Quorum Sensing” (QS) mechanisms of the host bacteria itself is out of the scope of this work, it could provide a possible explanation for why the bacteriophages would act in the plaque titration assay and not in the 96-well plate (Waters and Bassler, 2005). Thereby, in the plaque assay, titration *L. hilgardii* could reach the required cellular density to activate QS mechanisms and, therefore, the bacteriophage would have used the self-inducers to start lysis, while in the 96-well plaque, the phages remained under the lysogenic cycle. However, genetic assays would be needed to prove the existence of receptors or self-inducer molecules shared by *L. hilgardii* and its specific bacteriophage. If this were not proved, it would be necessary to study other factors related to the host bacteria growth. Another question that remains to be addressed is the difference between the ability of different phages to capture the same host bacteria when immobilized on a substrate (Double Layer Agar) or in a liquid media (Calendar, 2006; Hosseinidoust et al., 2011a, b). Additional research needs to be conducted in order to fully characterize the observed phenomena in wine of decreasing infection of phages in a liquid environment in contrast to a solid one.

Finally, bacteriophages that infected *O. oeni* showed an inhibitory effect on the growth of their host bacteria. Control curves had the same behavior as those of *L. plantarum*, with a quicker growth stabilization observed in red wine than in musts. Both isolated phages, Tin1*Oo* and Mos1*Oo*, showed a certain infective power. Nevertheless, the difference in growth between the control well and those with phage dilutions was significantly bigger in musts, which indicates that the host bacteria found must conditions more suitable to growth, promoting the infectious effect of the phage. The effect of these bacteriophages could not be compared with sulfur dioxide, since the bacteria growth was still much lower when the chemical compound was applied. Consequently, since phages showed a lytic effect on the host bacteria, it would only be necessary to increase the phage concentration to obtain a growth inhibition comparable to the sulfurous action. Results of the molecular analysis of *O. oeni* have established direct relationships between different bacteria strains and their bacteriophages. In 2005, the first genome sequence of *O. oeni* strain PSU-1 was published, and currently, information is available on the complete genome of 13 different strains. It was discovered that the main differences between the ORF (*open reading frame*) contents of bacteria strains are due to the presence of multiple bacteriophage sequences located at different points. At least four lysogenic phages can be integrated as complete elements or fragments in the *O. oeni* genome associated with the transfer of RNA binding sites (Borneman et al., 2012, 2013). The study of the conditions suitable for the activation of the lytic cycle of phages integrated in the bacterium genome could enhance the inhibitory effect on its growth.

The best results obtained in this work were those for bacteriophages that infected *L. plantarum*, whose effects were similar to sulfurous addition, followed by those of *O. oeni*, which showed less inhibition than obtained with SO_2_, while phages that infected *L. hilgardii* did not display any infective power under the experimental conditions. Although preliminary observations were obtained in this research, the presence of bacteriophages in the three grape varieties used highlights the potential application of a “phage cocktail” combining several infectious agents against a specific bacteria or different species or pollutants that require elimination in combination with a natural antioxidant. The fact that phages from the same family are able to infect different species allows savings to be proposed in the production of cocktails, in which the minimum number of bacteriophages could be used to prevent contaminating bacteria growth in the process of winemaking in a single treatment. In order to reduce the likelihood of resistant bacteria, cocktails could be designed that contain phages that use different receptors. In that way, if the mutant generated resistance to one of the phages in the cocktail, it would remain sensitive to the others. Currently, the use of lytic bacteriophages is a growing alternative for the biological control of pathogens in food and the food industry (Hermoso et al., 2007; García et al., 2008; Spricigo et al., 2013; Sulakvelidze, 2013). In the present study, the objective was not to remove pathogen microorganisms but those that cause losses in wine quality, such as lactic acid and acetic bacteria. Thus, the application of phage cocktails made by specific bacteriophages for every grape variety, vinification stage, and contaminating bacterium, together with a natural antioxidant, could be proposed in further studies at a larger scale.

## Data Availability Statement

All datasets generated for this study are included in the article/supplementary material.

## Author Contributions

GC-B, JM, and MR-C contributed to the conception and design of experiments. MR-C, MR-M, GC-B, and JM performed the experiments. MR-C, MR-M, GC-B, JM, and JC analyzed the data. MR-C and GC-B contributed to writing the manuscript. MR-M revised the English. All authors critically revised the manuscript before submission.

## Conflict of Interest

The authors declare that the research was conducted in the absence of any commercial or financial relationships that could be construed as a potential conflict of interest.
